# Neuro-Behçet’s masquerading as progressive bulbar palsy: a case report and literature review

**DOI:** 10.1177/2054270419834841

**Published:** 2019-03-25

**Authors:** Theodoros Paschalis, Noor M Shami, Amit KJ Mandal, Constantinos G Missouris

**Affiliations:** 1 Department of Medicine, University of Cambridge School of Clinical Medicine, Cambridge CB2 0SP, UK; 2 Department of Medicine, St George’s Medical School, Nicosia, 2408, Cyprus; 3 Departments of Medicine and Cardiology, Wexham Park Hospital, Frimley Health NHS Foundation Trust, Berkshire, SL2 4HL, UK; 4 Departments of Medicine and Clinical Cardiology, University of Cyprus Medical School, Nicosia, 2029, Cyprus

**Keywords:** neuroimaging, brain stem/cerebellum, clinical, neurology

## Abstract

In patients with progressive bulbar palsy without an obvious cause, there should be a high index of suspicion for the potential diagnosis of Neuro-Behçet’s Disease, even in the absence of the acute classical peripheral manifestations of Bechet’s Disease, with emphasis in prompt diagnosis using ‘The International Criteria for Behçet’s Disease’ and rapid, effective treatment in order to improve outcome.

## Introduction

Bechet’s Disease is a rare condition that may lead to a heterogeneity of neurological, vascular, gastrointestinal, renal and dermatological manifestations. We report a patient presenting with uncommon neurological findings, including progressive bulbar palsy, who was subsequently diagnosed with Behçet’s Disease.

## Case report

A 27-year-old previously fit man presented acutely to our hospital Emergency Department with sudden onset of diplopia, dysarthria, facial drooping and vomiting. This was preceded by a week of general malaise with progressively worsening headache and nausea. Two years earlier, he complained of recurrent oral and genital ulcers for which he had been prescribed oral antibiotics.

On examination, he was noted to have fever of 37.9℃, nuchal rigidity, mild right upper and lower limb weakness (Power 4/5), right upper facial nerve palsy and right abducens nerve paresis. There were no other neurological abnormalities of note, and the rest of the clinical examination was otherwise unremarkable.

**Figure 1.**
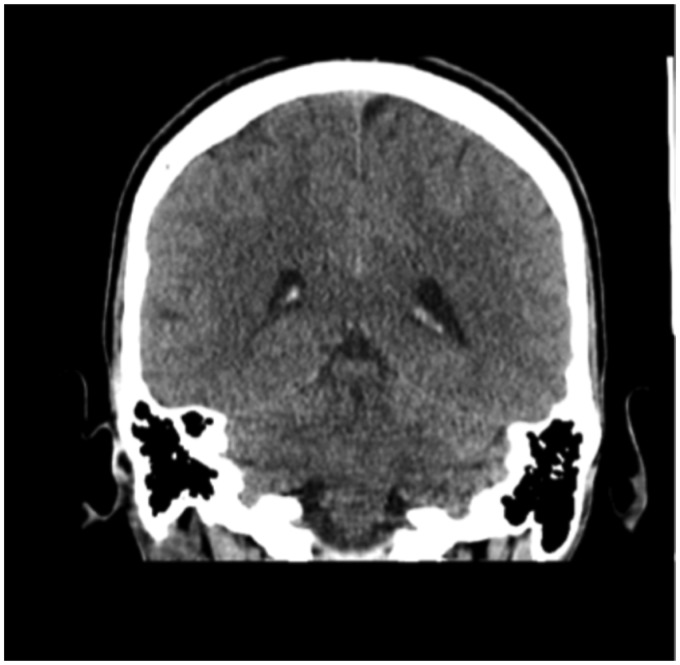
CT brain showing normal appearances.

The initial investigations included mildly raised inflammatory markers with a C-reactive protein of 23 mg/dL and an erythrocyte sedimentation rate of 29 mm/h. The CT brain was unremarkable (Figure 1).

**Figure 2.**
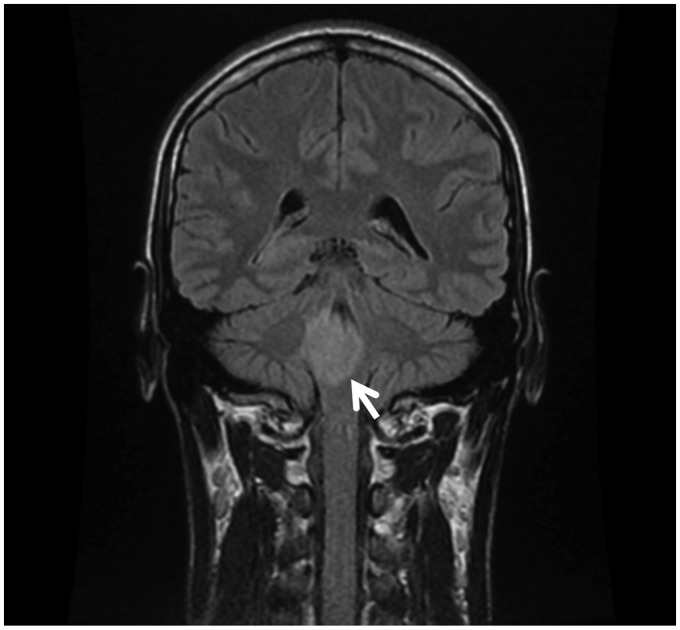
MRI brain showing a large ring enhancing lesion of the brainstem (arrow).

T2-weighted brain MRI scan was then undertaken, and this revealed a large lesion involving the pons and the medulla, with mild mass effect and ring enhancement (Figure 2).

Initial blood cultures grew *Streptococcus parasanguis* and *Streptococcus mitis* in one out of two bottles. Lumbar puncture sample was clear and colourless. Cerebrospinal fluid analysis revealed a raised cell count with a 95% lymphocytosis (240 × 106/L) and raised protein level at 1.41 g/L, but no organisms or oligoclonal bands were detected.

Based on the above results, the patient was empirically treated with intravenous Ceftriaxone, Vancomycin, Meropenem, Aciclovir and Amphotericin B. Despite broad spectrum pharmacotherapy, the patient deteriorated over the subsequent 72 hours with the development of dysarthria, dysphagia coupled with poor pharyngeal sensation and ataxic gait, in keeping with progressive bulbar dysfunction. Due to poor respiratory effort and declining consciousness, he required intensive care unit admission for tracheostomy and invasive ventilation. Given the previous history of oro-genital ulceration, a presumptive differential diagnosis of Neuro-Behçet’s Disease was made and the earlier positive blood cultures (in the context of repeated sterile blood cultures thereafter) were felt likely to be contaminants. The patient was transferred to a tertiary centre Neuro intensive care unit for specialist management. Prompt initiation of aggressive intravenous immunosuppression with methyl-prednisolone and cyclophosphamide resulted in dramatic clinical improvement, allowing respiratory weaning after 72 hours.

**Figure 3.**
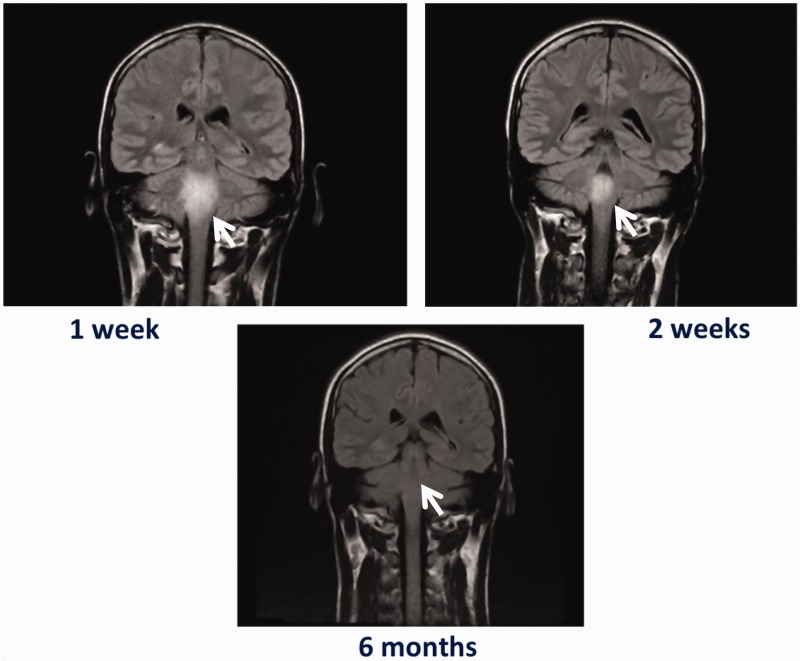
Staged T2-weighted MRI scans showing a significant interval improvement in the intracranial appearance, with almost a complete resolution of the signal abnormality.

Six months after the initial presentation, he remains asymptomatic with no neurological deficit, having completed a six-month regimen of oral methotrexate and prednisolone. There was a parallel resolution of radiological abnormalities (Figure 3).

## Discussion

Behçet’s Disease was first described by a Greek ophthalmologist Benediktos Adamantiades in 1930 as a syndrome of genital ulcers, arthritis and ocular signs. The name was coined after the Turkish dermatologist who described the typical triad of symptoms of aphthous oral and genital ulcers and recurrent uveitis in 1937.^1^ The involvement of many other systems has since been reported, including neurological, vascular, gastrointestinal, renal and dermatological manifestations. The aetiology of the above condition remains unknown; however, it is considered that Behçet’s Disease is a non-specific, multi-organ vasculitis, affecting vessels of different sizes.^1^ Typically, it affects patients in their second to fourth decade of life, rarely children and adults over 55 years of age and is commoner in men in the Mediterranean region but in women in the Far East.^2^ It is believed to have both an environmental and a genetic component. It is speculated that an auto-inflammatory reaction occurs following bacterial, viral or pollution exposure, in individuals with genetic susceptibilities. In particular, gene HLA-B*51 has shown an association with Behçet’s Disease, having been found positive in over 60% of patients with ocular involvement.^3^ However, the prevalence of the HLA-B*51 gene in patients with Behçet’s Disease appears to be related to the patient’s ethnic background. Despite the worldwide distribution of the disease, it is evidently more prevalent in countries along the ‘Silk Road’, an ancient trading route spanning from China to the Middle East, being most common in Turkey (421/100,000).^2^ In addition to that, gene HLA-B*51 is found more commonly in these populations of patients than in Caucasian populations, favouring a genetic aetiology for Behçet’s Disease.^4^

Despite the increasing knowledge around Behçet’s Disease, diagnosis is purely clinical, in the absence of any relevant biological test. Various classification criteria have been developed over the years. The International Study Group for Behçet’s Disease proposed in 1990 a set of criteria for clinical trials of Behçet’s Disease, which were used in the diagnosis of Behçet’s Disease.^1,2^ Using these criteria, a diagnosis was made based on the presence of three or more symptoms of Behçet’s Disease, one of which had to be oral ulceration. The commonness of oral lesions in other diseases, for example Crohn’s Disease, led to criticism of the above criteria’s diagnostic role. Subsequent reassessment led to a symptoms-scoring system, attributing two points for each of the major symptoms, as described by Behçet, and one point for every other positive symptom (neurological, skin, vascular, pathergy test), where a score of four or more is considered diagnostic for Behçet’s Disease.^5^ Raised C-reactive protein, erythrocyte sedimentation rate and other acute phase reactants do not confer any specificity for the diagnosis of Behçet’s Disease; however, these findings should prompt active investigation, having been found raised in Behçet’s patients with active disease compared to inactive.^1,2^ Nonetheless, diagnosing Behçet’s Disease remains difficult because the time between the manifestation of the cardinal symptoms can vary considerably.

Neurological involvement is termed Neuro-Behçet’s Disease and more frequently affects male patients within 10 years from the onset of the disease.^6^ Neuro-Behçet’s Disease seems to have its own geographical distribution, being more prevalent in Caucasian populations in ‘Western’ countries and occurs alongside the classical symptoms of Behçet’s Disease.^7^ In a study of 200 patients with Neuro-Behçet’s Disease, presentation of neurological symptoms varied widely but was divided into parenchymal and non-parenchymal disease according to site of CNS involvement, with the former being more common.^6^ In patients with parenchymal disease, the most common presentation was with brainstem involvement and less frequently with cerebral hemisphere involvement. Clinically, the most common symptoms and signs of parenchymal Neuro-Behçet’s Disease were bilateral pyramidal signs, unilateral hemiparesis, behavioural changes and headache. Presentation with brainstem signs including bulbar signs and ophthalmoplegia, fever and meningeal signs were some of the rarer manifestations. Progression of the neurological symptoms was also varied, with a sudden onset (attack) being more frequent. The same study reported that the cerebrospinal fluid of patients with parenchymal disease was typically pleocytotic with high protein content. In non-parenchymal disease, patients typically show intracranial hypertension due to Dural Sinus Thrombosis, with the most common signs being papilledema and sixth nerve palsy, and most patients reporting headache and high fever during acute attacks. cerebrospinal fluid in patients with non-parenchymal disease was found to be unremarkable apart from its raised pressure. MRI scans were shown to be more useful in identifying brain lesions than CT scans, with the basal ganglia or the brainstem being the most frequently affected areas during an acute attack. Mass effect with contrast enhancement was seen in some of the cases.

Interestingly, Noel et al.^8^ discussed a series of 23 cases which were sub-classified as pseudotumoural parenchymal Neuro-Behçet’s Disease. This rare form (1.8%) of parenchymal Neuro-Behçet’s Disease differs from classical parenchymal Neuro-Behçet’s Disease due to its presentation with a single intracerebral mass-like lesion, often in the capsulothalamic area. Although our case can be considered classical parenchymal Neuro-Behçet’s Disease based on the location of the lesion in the brainstem, it resembles pseudotumoural parenchymal Neuro-Behçet’s Disease with a single space-occupying lesion, seen on MRI but not on CT and the life-threatening presentation in an undiagnosed patient. This point is reinforced by the case presented by Kir et al.,^9^ in which the patient had similar neurological manifestations, with the addition of right-sided ptosis, third cranial nerve palsy and unreactive, dilated pupil. There was also a history of genital and oral ulcers and a similar approach to investigations was taken as in our case. A right-sided capsulothalamic lesion was identified on MRI, classifying this case as a pseudotumoural parenchymal Neuro-Behçet’s Disease case. Despite the variability in clinical and radiological presentation, the patient received the same treatment regime as in our case; however, no follow-up data were provided.

Treatment of Behçet’s Disease is not straightforward, partly because of the variability of its manifestations and partly because of the absence of a single effective therapeutic agent. The aim of treatment is symptomatic relief and prevention of irreversible damage.^1,2^ The approach taken will depend on the patient’s symptoms and be based on the involvement of particular systems, making the involvement of a multi-disciplinary team very important. Current guidelines for the treatment of Behçet’s Disease are based on recommendations by the European League Against Rheumatism, which were formulated based on evidence-based treatments of some manifestations of Behçet’s Disease (colchicine in mucocutaneous manifestations^2^) and the expert opinions of an multi-disciplinary team for the remaining systems involved in the disease.^10^ The treatment of Neuro-Behçet’s Disease falls in the latter category, where no controlled data guide the management. In patients with parenchymal involvement, high doses of three to seven IV corticosteroid pulses are given during acute attacks, with a three-month oral maintenance regime. Prevention of recurrence is achieved by the use of immunosuppressive agents such as Azathioprine or Cyclophosphamide in more severe cases.^10^ Biological agents targeting tumor necrosis factor-α were also shown to be effective in the treatment of Cyclophosphamide-resistant Neuro-Behçet’s Disease.^11^ Finally, studies suggest avoiding Ciclosporin A in Neuro-Behçet’s Disease because of its potential neurotoxicity which can potentiate neurological involvement, unless treatment of eye involvement necessitates it.^12^

## Conclusion

Our case report clearly demonstrates that in patients presenting with neurological symptoms, in particular progressive bulbar palsy, for which no obvious cause is found, there should be a high index of suspicion for the potential diagnosis Neuro-Behçet’s Disease even in the absence of acute classical peripheral manifestations of Behçet’s. Emphasis is laid on the importance of prompt diagnosis using the revised ‘International Criteria for Behçet’s Disease’ and rapid, effective treatment in order to improve outcome of this condition which invariably carries a poor prognosis.

## Declarations

### Guarantor

AKJM.
